# MiRIAD update: using alternative polyadenylation, protein interaction network analysis and additional species to enhance exploration of the role of intragenic miRNAs and their host genes

**DOI:** 10.1093/database/bax053

**Published:** 2017-08-01

**Authors:** Ludwig C. Hinske, Felipe R. C. dos Santos, Daniel T. Ohara, Lucila Ohno-Machado, Simone Kreth, Pedro A. F. Galante

**Affiliations:** 1 Department of Anaesthesiology, University Hospital of the Ludwig-Maximilians-University Munich, Munich, Germany; 2 Centro de Oncologia Molecular, Hospital Sírio-Libanês, São Paulo SP 01308-060, Brazil; 3 Inter Unidades em Bioinformática, Instituto de Matemática e Estatística, Universidade de São Paulo, São Paulo, SP, Brazil; 4 Health System Department of Biomedical Informatics, University of California San Diego, La Jolla, CA 93093, USA

## Abstract

MicroRNAs have established their role as potent regulators of the epigenome. Interestingly, most miRNAs are located within protein-coding genes with functional consequences that have yet to be fully investigated. MiRIAD is a database with an interactive and user-friendly online interface that has been facilitating research on intragenic miRNAs. In this article, we present a major update. First, data for five additional species (chimpanzee, rat, dog, cow and frog) were added to support the exploration of evolutionary aspects of the relationship between host genes and intragenic miRNAs. Moreover, we integrated data from two different sources to generate a comprehensive alternative polyadenylation dataset. The miRIAD interface was therefore redesigned and provides a completely new gene model representation, including an interactive visualization of the 3′ untranslated region (UTR) with alternative polyadenylation sites, corresponding signals and potential miRNA binding sites. Furthermore, we expanded on functional host gene network analysis. Although the previous version solely reported protein interactions, the update features a separate network analysis view that can either be accessed through the submission of a list of genes of interest or directly from a gene’s list of protein interactions. In addition to statistical properties of the submitted gene set, the interaction network graph is presented and miRNAs with seed site over- and underrepresentation are identified. In summary, the update of miRIAD provides novel datasets and bioinformatics resources with a significant increase in functionality to facilitate intragenic miRNA research in a user-friendly and interactive way.

**Database URL:** http://www.miriad-database.org

## Introduction

MiRNAs are well-known as small molecules that are involved in controlling regulatory networks of the gene expression (1). Interestingly, most (e.g. 61.5% for human and 66.2% for mouse) miRNA genes are positioned within protein-coding genes in vertebrates (2, 3). These miRNAs are called intragenic miRNAs and their enclosing genes ‘host genes’. Accumulating evidence suggests that this special relationship of genomic colocalization between an intragenic miRNA and its host gene is of biological relevance. Negative feedback loops of intragenic miRNAs regulating their host genes have recently been described, ranging from first-order (i.e. direct) negative feedback (4–6) to indirect feedback loops (2, 7, 8).

Using a myriad of data from different sources and databases focused on the analysis of intragenic miRNAs (3, 9–13), we and others have found further functional implications of intragenic miRNAs and their host genes. Recent research suggests evolutionary implications of intragenic miRNA development (14, 15), yielding that novel miRNAs seem to benefit from intragenic colocalization by utilizing existing regulatory circuitries of their host genes (14). Furthermore, increasing evidence highlights the importance of the role of alternative polyadenylation (APA) to characterize the relationship between intragenic miRNAs and their host genes (5, 6). These novel discoveries prompted us to develop a major update of the miRIAD database and interface to account for these new aspects of intragenic miRNA–host gene relationship.

In this article, we provide a detailed description of the updated version of miRIAD. In its first version, miRIAD integrated genomic data for five species to classify miRNAs into intergenic, intronic and exonic, allowing easy identification of intragenic miRNAs and host genes (3). In the updated version, miRIAD contains five additional species (chimpanzee, rat, dog, cow and frog). Among other changes, it was redesigned to include APA information from two different sources (16, 17) for 8 of 10 included species (human, rhesus, chimpanzee, mouse, rat, dog, opossum and chicken). To maximize utility of these new data, the gene model visualization was completely redesigned to implement interactive vector graphics. Interaction network analysis functionality was added to allow evaluation of a set of genes (e.g. gene signatures) with respect to host gene over- or underrepresentation, visualization of protein interactions with respect to intragenic miRNA targeting and identification of over- or underrepresented miRNA target sites in a network. We also show, how to use the new functionality to derive hypotheses about the relationship between a host gene (AKT2) and its intragenic miRNA (hsa-miR-641). To the best of our knowledge, miRIAD is the first public resource to allow these analyses to investigate the role of intragenic miRNAs.

## Materials and methods

### MiRIAD construction and integration of additional species

**Figure 1.**
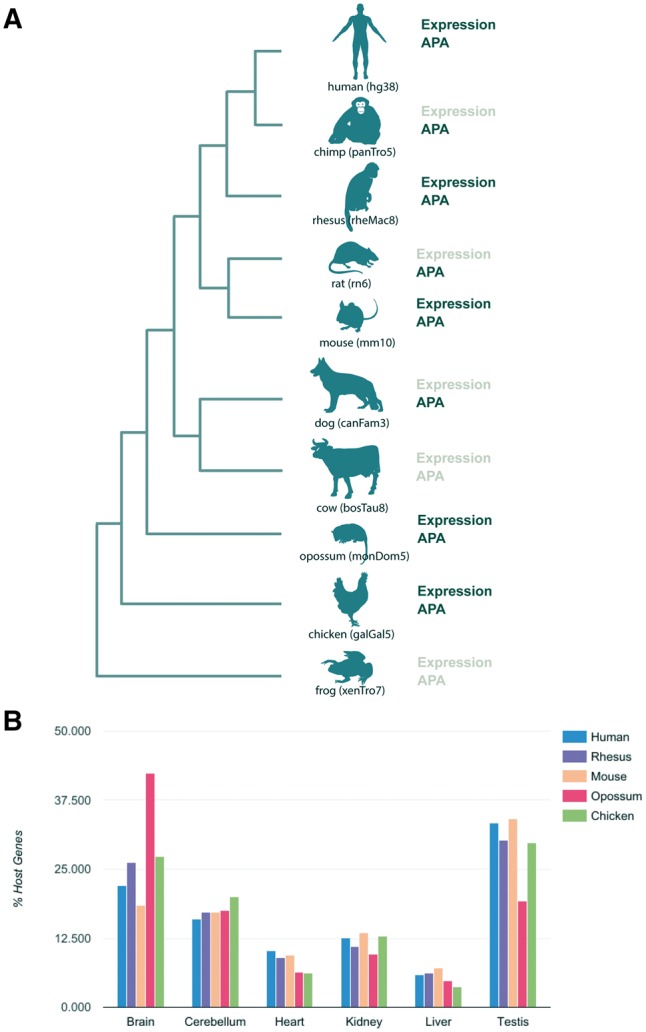
Summary of species in the miRIAD and host genes expression. (**A**) Species present in the miRIAD. (**B**) Host genes expression in six tissues.

Selection of species to be integrated in miRIAD was based on several factors. First, we required the availability of high quality genome assemblies and a good RefSeq coverage. Second, we searched for available polyadenylation, gene and miRNA expression data. Construction of the miRIAD database was performed with the newest genome assemblies (human: hg38/GRCh38, rhesus: rheMac8, chimp: panTro5, frog: xenTro7, cow: bosTau8, opossum: monDom5, rat: rn6, chicken: galGal5, dog: canFam3 and mouse: mm10; Figure 1A) and mirBase version 21 (12), as described in (3). Coding gene and miRNA expression was calculated from RNA-Seq data from Brawand *et al.* (17), Gene Expression Omnibus (GSE30352). RNA-Seq data processing was carried out as previously described (3).

### APA information for eight species

We combined APA data from a previously published dataset from (16) (human, rhesus, dog, mouse and rat) with APA information that we derived from processing the dataset obtained by Brawand *et al*. (17). Poly(A) coordinates from Derti *et al.* were mapped to the respective current genome assemblies using the liftOver tool provided by the Genome Browser from the University of California Santa Cruz (UCSC) (18). Identification of APA sites from RNA-Seq data from Brawand *et al.* was carried out as follows. After data preprocessing [for details see (3)], reads were filtered for those starting or ending with at least four untemplated ‘A’s or ‘T’s. Reads with an extremely high A/T/N-content were ignored (cut off ratio was set to 0.8). Potential APA sites were considered, if they (i) mapped to a untranslated region (UTR)-annotated region based on RefSeq and (ii) were supported by at least two independent reads. APA sites within 40 nucleotides were considered to be a single APA site. For benchmarking, expressed sequence tags based alternative poly(A) site information from APADB (19) were downloaded for human and mouse. For human, APA site information was converted to hg38 using the liftOver tool (18). Only APA sites mapping to RefSeq UTR models were considered.

### Target predictions and protein interaction network

The protein interaction network feature visualizes relationships between gene products as an interactive scalable vector graphics (SVG) image. Using an enrichment–calculation-based target prediction network score, it may also help to identify miRNAs relevant for regulation of this network, which yet lacks experimental support. Target predictions are based on canonical seed matching used by Targetscan on the 3′-UTR sequences of protein-coding RefSeq transcripts (10). In brief, 3′-UTRs are scanned for base complementarity to Bases 2–7 of the mature miRNA sequences (seed region). Hybridization energy between miRNA and UTR sequence was calculated using the Vienna RNA library (20). The impact of a miRNA on a set of genes is quantified as follows: First, the probability of random occurrence of a given seed sequence is calculated by $P(S)=\prod_{i=1}^{n}P(N_{i}|D)$, where *S* = seed sequence, *n* = length(*S*), *N_i_* = *i*th nucleotide of *S*, *D* = nucleotide distribution.

The probability that this sequence occurs at least *r* times in a random sequence of length *N* (UTR sequences for each gene in the network) is given by:

$$P(x_{t})=\;(1-\sum_{i=0}^{r-1}\left(\left(\frac{L_{x}}{i}\right)*P{\left(S\right)}^{i}*{\left(1-P(S)\right)}^{L_{x}-i}\right)),$$

where *L_x_* = (length of 3′-UTR of element *x_t_*) − (length of seed sequence *n*) + 1, *r* = desired minimum number of occurrences, miRIAD is using *r* = 1.

The expected number of genes containing seed-matching sites *E*(*X_t_*) in the network *X* can then be estimated by the sum of probabilities for each gene *x*.

$$E(X_{t})=\sum_{x_{t}\in X}P(x_{t})$$

This number of expected random target genes in the network can be compared with the observed number of genes with seed matches. Statistical evaluation is possible using Fisher’s exact test. The score reported in miRIAD equals the log-odds ratio, given by:

$$\text{Score}(X\;|\;S)=\;\;\text{log}\;(\frac{\frac{E(X_{t})+\;O(X_{t})}{E(X_{t})}*\;\frac{E(X_{n})+\;O(X_{n})}{E(X_{n})}}{\frac{\left|X\right|}{E(n)}}).$$

## Results

### Database statistics

The current version of miRIAD contains 10 species, with a total of 284 374 protein-coding genes and 7369 miRNAs. In total, 61.5% of human and 66.2% of mouse miRNAs are intragenic. Expression data for miRNAs as well as for mRNAs are available for six organs (brain, cerebellum, heart, kidney, liver and testis) from human, mouse, rhesus, opossum and chicken (Figure 1A). Investigating the distribution across tissues in human, we found that host genes of intragenic miRNAs are predominantly expressed in neuronal tissue and testis across all organisms (Figure 1B). We were able to extract APA information for 8 of 10 species in miRIAD (Figure 1A). According to our database, 94.6% of human host genes have annotated APA sites, which is more than expected compared with 83% of all human genes (*P* value = 4.6e-38, Fisher exact test). Similarly, 92.3% of murine host genes and have annotated APA sites (72% of all murine genes). This relationship is true with varying degrees for chicken (18% of host genes, expected 11%, *P* value = 3.5e-4), rat (78 vs 59%, *P* value = 5e-06), rhesus (80 vs 65%, *P* value = 2e-06) and chimpanzee (45 vs 28%, *P* value = 3e-09). We did not find significant differences in dog (71 vs 69%, *P* value = 0.68) and opossum (11 vs 8%, *P* value = 0.26). Summarized statistics are available in Table 1. We used the previously published database APADB to benchmark APA sites for mouse and human included in miRIAD (19). APA site information for a total of 14 143 human and 13 472 murine genes was compared. miRIAD includes 29 349 of the 34 753 events registered in APADB mappable to our UTR models (84.5%). Similarly, 82% of murine APA sites were covered by miRIAD (20 826 of 25 323).

**Table 1.** Summarized statistics for miRNAs and host genes

Organism (genome assembly)	miRNAs (total)	Total number of genes	Total number APA sites	Intragenic miRNAs			Intergenic miRNAs	Host genes	Host genes with APA sites	APA sites in host genes	Number of predicted target interactions	Number of protein interactions
				intronic (sense in %)	exonic (sense in %)	all (sense in %)						
*Homo sapiens* (hg38)	1881	20 204	206 656	988 (82.4%)	169 (81.1%)	1157 (82.2%)	724	984	949	16 921	22 930 745	7 553 352
*Pan troglodytes* (panTro5)	628	33 003	39 860	280 (86.8%)	22 (45.5%)	302 (83.8%)	326	253	115	1066	4 230 060	8 182 491
*Mulatta macaca* (rheMac8)	486	28 336	101 385	214 (83.2%)	21 (57.1%)	235 (80.9%)	251	201	161	1515	3 804 170	11 339 842
*Mus musculus* (mm10)	1187	36 058	124 297	644 (87.4%)	142 (81.0%)	786 (86.3%)	401	649	599	6377	12 405 027	11 090 779
*Rattus norvegicus* (rn6)	485	32 387	48 198	153 (73.2%)	45 (62.2%)	198 (70.7%)	287	143	111	725	4 281 941	12 781 450
*Bos taurus* (bosTau8)	811	27 121	NA	396 (79.5%)	45 (68.9%)	441 (78.5%)	370	356	NA	NA	4 371 894	9 953 188
*Canis familiaris* (canFam3)	513	24 782	79 089	192 (81.3%)	25 (12.0%)	217 (73.3%)	269	171	121	982	2 025 226	9 551 812
*Gallus gallus* (galgal5)	709	25 610	6561	350 (87.7%)	43 (58.1%)	393 (84.5%)	316	353	63	217	4 366 456	2 845 210
*Monodelphis domesticus* (monDom5)	460	33 101	5299	165 (89.1%)	2 (0.0%)	167 (88.0%)	293	131	14	56	6 056 838	11 585 890
*Xenopus tropicalis* (xenTro7)	209	23 772	NA	61 (60.7%)	0	61 (60.7%)	148	46	NA	NA	1 066 564	0
	7369	284 374	611 345	3344	481	3825	3385	3287	2133	27 859	65 538 921	84 884 014

NA, not available

### Interactive structural representation of UTR, miRNA and host gene relationship

**Figure 2.**
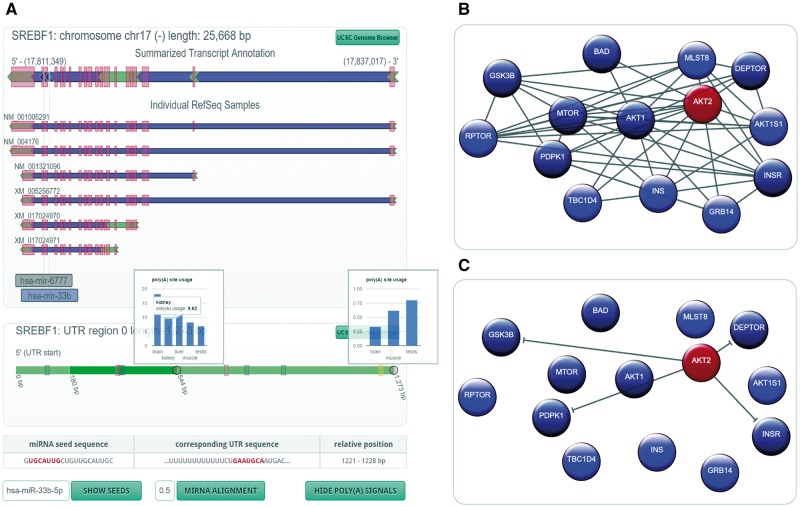
miRIAD representation for a host gene, its intragenic miRNAs, protein–protein interaction (PPI) data and an intragenic miRNA target. (**A**) Genomic representation (including polyadenylation information) for a host gene (SREBF1) and its intragenic miRNAs (hsa-mir-6777 and hsa-mir-33b). (**B**) PPI network for AKT2; (**C**) gene targets for hsa-miR-641, which is an intragenic miRNA for AKT2.

The representation of structural properties of a host gene and its intragenic miRNA is of great importance when investigating their relationship (2, 14). In the new miRIAD-version, we developed a representation based on interactive SVG to visualize the gene structure, highlighting exonic, intronic and UTRs (Figure 2A). It contains a summarized representation, in which region information is merged, followed by individual RefSeq transcripts of the gene of interest. The positions of intragenic miRNAs are shown in the summarized transcript and relative to individual transcripts. This allows the researcher to check for transcripts devoid of the intronic miRNA, proximity to upstream exons as an indicator of cotranscription or organization of miRNA genes in mirtrons. Figure 2A shows the gene model representation of SREBF1 with its intronic miRNAs *miR-6777* and *miR-33b*. The latter is highlighted in blue to indicate that the host gene has at least one seed-matching site within its 3′-UTR.

In addition to structural properties of the gene, the organization of the 3′-UTR further characterizes the relationship between an intragenic miRNA and its host gene. We therefore included a novel representation of 3′-UTR variants based on published and self-constructed APA information. Segmentation of the UTR by APA sites is symbolized by alternating shades of green. If the user moves the mouse cursor over an intragenic miRNA highlighted in blue, the position of the seed within the UTR will show up. In the case of SREBF1 and hsa-miR-33b, the seed site is located on the 3′ extremity of the transcript with at least one isoform without this seed-matching site. A click on the button ‘show poly(A) signals’ reveals canonical polyadenylation signals, in this case indicating that there might actually be another APA site that has not yet been described. To support investigation of differential miRNA targeting, APA site utilization across tissues can be visualized where available. A click on a gray circle in the 3′-UTR will open utilization information for this site.

Additionally, the ‘show seeds’ option allows the identification of seed-matching sites for any miRNA. If no seed matches are found, the user can choose ‘miRNA alignment’ to search for regions of high similarity to the mature miRNA sequence, helping to identify non-canonical miRNA binding sites. Clicking on a potential miRNA binding site in the UTR (either yellow for seed sites or gray for non-canonical sites) will show the sequence of the miRNA and the sequence of the region of interest in the 3′-UTR (Figure 2A).

Each of the 3′-UTR model representation displays a button on the right top corner that will open the UCSC Genome Browser (18, 21) for the specific UTR region or the full gene model. In this way, a plethora of additional information can be gained, such as evolutionary conservation, without sacrificing simplicity of miRIAD interface usage.

The interactive, visual representation of the gene model is followed by expression information of the gene across the tissues cerebellum, brain, heart, liver, kidney and testis, as well as a figure correlating the expression of the intragenic miRNA with the host gene across these tissues, providing Spearman’s rank correlation coefficient and a *P* value. These figures help to rapidly identify tissue specificity, as well as coregulation (indicated by high correlation of expression). An interesting example is that of MAP2K4 and intragenic miRNA ‘hsa-miR-744’, in which both miRNA transcripts (hsa-miR-744-3p and hsa-miR-744-5p) correlate extremely well with their host gene’s expression. Similar to the gene view, the miRNA view yields the structural representation of the miRNA gene, expression across tissues and correlation graphics with their host genes.

### Filtering of targeting miRNAs and protein interactions

Although some decades ago, research was focusing on the exploration of single genes only, evaluation of protein interactions and regulation through miRNAs has become increasingly important. Although both protein interaction and target prediction information were already available in the first version of miRIAD, it now includes more data and supports filtering of these. Targeting miRNAs for example can be filtered by score or by name, in case the user wants to check a specific location for a miRNA target interaction or just wants to find miRNAs with a high binding probability. Differential miRNA targeting can be assessed by identifying miRNAs that bind only to a specific APA isoform through filtering for a specific poly(A) index. A click on the miRNA symbol will highlight the seed match(es) within the UTR of the gene (Figure 2A). Also, the list of miRNAs can be significantly reduced by filtering for the tissue of maximum expression. This is especially useful, when looking for potential regulators of a gene that shows strong tissue specificity.

Similarly, genes whose products interact with the gene of interest can be filtered by gene name, score and type of interaction (for STRING), evidence of binding (e.g. two hybrid system or direct interaction for BIND) and by data source [Bind (22), STRING (23), HPRD (24) and BioGRID (25)].

If the gene of interest contains intragenic miRNAs, information on interacting proteins that are potentially targeted by this miRNA will appear. This allows the user to estimate the impact of the intragenic miRNA on the host gene better. The filtered selection of interacting genes can then be submitted to the newly introduced network analysis view for extended evaluation.

### Network view: analysis of complex interactions

It is known that intragenic miRNAs have a special impact on their host genes’ surrounding network (2, 14). We therefore implemented an algorithm that helps identify miRNAs relevant for networks of genes. If a researcher identifies a set of interesting genes, e.g. a cancer gene signature, it might be of great interest, whether host genes are over- or underrepresented in this gene signature, how these genes interact with one another and if there are miRNAs relevant to this gene signature as a whole. A miRIAD query with a preceding colon followed by the gene symbols of the signature (separated by spaces) will load the network view to help answer these biologically relevant questions. First, statistics on the number of host genes in the submitted gene list (including an estimative of the significance of over-/underrepresentation), their intragenic miRNAs (if any) and the most relevant properties of each relationship (same strand, seed site within host UTR) are shown. The most central part is the network representation (Figure 2B and C), which visualizes regular genes (blue), host genes (red) and protein interactions between them. Network nodes can be rearranged by the user for better visualization, and mouseover will highlight all nodes with direct interactions, which makes it easy to identify hubs in large networks. Interactions can be filtered by score or data origin. Also, if the network contains host genes, interaction arrows can be replaced by predicted target interactions of the intragenic miRNA(s) (Figure 2C).

### Exploring the relationship between AKT2 and its intronic miRNA miR-641

*AKT2* hosts intragenic miRNA *hsa-miR-641* but the relationship between these two being largely unknown. The gene structure representation shows that miR-641 is located on the same strand as its host gene, and that it is positioned in the first intron. Although this fact *per se* might suggest coregulation, there are four (predicted) RefSeq transcripts that don’t include miR-641. Correlation between miRNA and host gene cannot be well-characterized, since miR-641 seems to be only expressed in neuronal tissue. Filtering AKT2’s interaction partners for STRING-reported interactions with a minimum score of 900 reveals the network in Figure 2B. MiRNA hsa-miR-637 ranks high in the list of miRNAs that potentially impact the network (score 1.34; targeted genes are dark-blue/dark-red). It is known to control the AKT-pathway (26). Interestingly, targets are very similar to hsa-miR-641 (Figure 2C), indicating a similar function for these two miRNAs. Moreover, miR-641 is only also found in chimp in our dataset, suggesting a relatively new evolutionary role. This example shows how miRIAD can be used to derive hypotheses about the relationship between a miRNA and its host gene.

## Discussion

Nowadays, in the era of large scale data generation in genomics and transcriptomics, it is essential to have powerful and user-friendly tools to mine the right information, to propose and to test hypotheses regarding the studied model. The special genomic colocalization of most vertebrate miRNAs intragenically is of great relevance and current studies have been revealing that the functional implications of this coupling extend beyond simple feedback regulatory mechanisms but seems to support miRNA evolution (2, 5, 14). This revelation expands the focus of research requiring tools to study intragenic miRNAs and genes in an evolutionary context. The new version of miRIAD was therefore extended to a total of 10 species, covering major phylogenetic branches. Statistics on APA show that significantly more host genes contain APA sites than would be expected. This is even true for chicken, the most distant specie investigated. Interestingly, dog and opossum, both being closer to human, don’t display this phenomenon. This discovery might be biased by the fact that genome annotation of dog and opossum is not as complete as other genomes but it may also be a starting point for the investigation of a potentially underlying biological principle.

These analyses are complemented by newly implemented data and functionality to accommodate complex data investigation, such as miRNA-host gene centered network analysis and visualization of APA with respect to miRNA binding sites. MiRIAD can now be used to derive interesting hypotheses about the relationship between a miRNA and its host gene. As it was illustrated for AKT2 and its intragenic miRNA miR-641, e.g. miRIAD allowed us to generate the hypothesis that miR-641 might control the AKT pathway in neuronal tissue in human and chimp. It also allows rapid identification of miRNAs that may bind to specific UTR regions or target only specific alternatively polyadenylated isoforms.

At this point, complete gene and miRNA expression and APA information is available only for 8 of 10 species. This is owed to fact that currently only Brawand *et al.* (17) provide a dataset that contains RNA sequencing information on miRNAs and mRNAs from the same individuals, across multiple species and tissues. However, we expect to be able to include additional datasets in future versions. We hope to provide additional poly(A) site information for frog and cow, as well as miRNA and mRNA expression data. Furthermore, miRIAD currently implements target predictions only through seed site matching, ignoring non-canonical sites. This strategy is necessary for the implementation of our model that quantifies the probability of a miRNA-network effect. However, miRIAD is an ongoing project and we are planning to present an extended model that includes non-canonical sites, tissue specificity and APA information in upcoming releases.

In summary, the new version of miRIAD adds important new data and functionality to enhance the exploration of the role of intragenic miRNAs through providing APA information and network analysis in the light of phylogeny.
